# Seasonal variation of glycated haemoglobin and estimated average glucose in temperate South Africa

**DOI:** 10.4102/jcmsa.v2i1.107

**Published:** 2024-11-14

**Authors:** Sinazo Ximbi, Bettina Chale-Matsau, Ashlin Rampul, Tahir S. Pillay

**Affiliations:** 1 Department of Chemical Pathology, Faculty of Health Sciences, University of Pretoria, Pretoria, South Africa; 2 National Health Laboratory Service (NHLS), Pretoria, South Africa; 3 Department of Chemical Pathology, Pathcare Laboratories, Durban, South Africa

**Keywords:** glycated haemoglobin, seasonal variation, estimated average glucose, diabetes mellitus, HbA1c

## Abstract

**Background:**

Glycated haemoglobin (HbA1c) is a useful biomarker for the monitoring and diagnosis of diabetes mellitus. In the northern hemisphere, there is evidence of seasonal fluctuations in HbA1c with values being higher in cooler months and lower in warmer months. In the southern hemisphere, there is one description from Australia. It is not known if this would also pertain in other temperate climates. We explored the relationship between seasons, HbA1c and estimated average glucose in South Africa.

**Methods:**

This was a retrospective analysis. HbA1c data were obtained from January 2016 to December 2020 from two different provinces with different climates. The mean HbA1c values and mean monthly temperatures were compared for each year.

**Results:**

We show seasonal variation for HbA1c and estimated average glucose (eAG) in both provinces (*p* < 0.01). The population was further subdivided and evaluated based on gender and age. Glycated haemoglobin was higher in cooler months and lower in the warmer months for all the data and for both the categories (age and gender; *p* < 0.01).

**Conclusion:**

South Africa shows demonstrable seasonal variation in glycated haemoglobin that needs to taken into account when managing diabetes mellitus.

**Contribution:**

Awareness of this seasonal effect may assist in more efficient use of HbA1c to monitor and diagnose diabetes mellitus and will assist in modifying therapeutic targets for achieving good glycaemic control.

## Introduction

Comparability of patient results is an important aspect of laboratory medicine. Laboratory results guide clinical management, and results from different laboratories need to be comparable. Technological developments in clinical chemistry have enabled standardisation of analytes and a marked reduction in analytical and post-analytical variability with the use of automated analysers.^1^ Standardisation of analyte measurements means that results will be uniform. Results will be traceable to a recognised reference material and will be uniform irrespective of the assay methodology. Glycated haemoglobin (HbA1c) is one such analyte which has benefitted from these technological advances. Current studies are focussed on the origin of pre-analytical variability, such as intra-individual differences, biological rhythms and seasonality demonstrated by biochemical analytes. Haemoglobin A1c (HbA1c) is the primary test for the assessment of glycaemic control for patients with both type 1 and type 2 diabetes; however, there are other criteria for the diagnosis of diabetes mellitus.^2^ The four criteria for the diagnosis of diabetes mellitus are random blood glucose ≥ 11.1 mmol/L with symptoms of diabetes or a hyperglycaemic crisis, HbA1c ≥ 6.5%, fasting plasma glucose of ≥ 7 mmol/L or a 2-h plasma glucose ≥ 11.1 mmol/L during an oral glucose tolerance test.^3,4^ HbA1c can also be used to monitor glycaemic control and to report estimated average glucose over a period of 2–3 months, which takes advantage of the 3–4 months’ approximate life span of erythrocytes.^5,6^

The goal of treatment for diabetes mellitus is to reduce the incidence of both microvascular and macrovascular complications, and to improve prognosis.^3,7^ In South Africa, one of the criteria in the diagnosis of diabetes mellitus is an HbA1c ≥ 6.5%, and the treatment targets of HbA1c < 7% as aligned with international norms.^8^ These are the recommendations from the Society for Endocrinology, Metabolism and Diabetes of South Africa (SEMDSA).^8^

According to some studies in the northern hemisphere regarding seasonality of HbA1c, HbA1c values are higher in cooler/winter months and lower in warmer/summer months.^2,7,9,10,11^ Studies in the southern hemisphere have been limited only to Australia.^2,12^ There are no published data from Africa or South Africa on the seasonal variation of HbA1c. To our knowledge, this is the first study to explore a relationship between seasonal changes and estimated average glucose (eAG).

## Research methods and design

This is an observational, cross-sectional study using HbA1c and eAG results generated from the National Health Laboratory Services, Tshwane Academic Division (NHLS, TAD) and Inkosi Albert Luthuli Hospital (NHLS-IALH), requested from the NHLS data archives. These laboratories are situated in two provincial regions: Durban, KwaZulu-Natal and Pretoria, Gauteng in South Africa. The NHLS-Tshwane Academic Division (Pretoria) and the NHLS-Inkosi Albert Luthuli Central Hospital (Durban) are attached to two tertiary hospitals in South Africa with an approximated radius of 600 km from each other and different climates. Durban is situated along the coast and has a subtropical climate, with warm, humid summers (November to March) and mild, drier winters (June–August). Winters are typically warm, with temperatures rarely falling below 10 °C. Pretoria, located inland at a higher elevation, has a continental climate with hot summers and cool, dry winters. Summers (November–March) are warm, with afternoon thunderstorms, while winters (June–August) are cool, with temperatures occasionally dropping to around 0 °C at night but warm during the day.

The HbA1c measurements from Durban (*N* = 424 017) contributed approximately 73% of the data points while those from Pretoria (*N* = 155 812) contributed approximately 26%. HbA1c measurements (*N* = 579 829) were analysed on EDTA-whole blood using high-performance liquid chromatography (HPLC) Bio-Rad Variant II Turbo (Gauteng) and Tosoh G8 HPLC automated (KwaZulu-Natal) analysers. These assays have an analytical imprecision (CVa) of < 5%, are National Glycohemoglobin Standardization Program (NGSP) certified and meet International Federation of Clinical Chemistry Working Group (IFCC-WG) analytical goals.^13^ The intra-assay CVs are 1.15% and 1.3%, respectively. Mean monthly temperatures were extracted from available meteorological information (South African Weather service; https://www.weathersa.co.za/). The mean HbA1c result and the mean monthly temperature were compared during each month of the year for the two provincial regions and for the data when combined.

Patient data were divided into two age groups, > 40 years and ≤ 40 years, according to gender (male and female) for the two regions and for all the data. The eAG for each sample was calculated using the formula:

(HbA1c*1.98)−4.29 [Eqn 1]

and reported in millimoles per litre (mmol/L).^5^ The mean eAG and the mean monthly temperature were also compared during each month of the year. There are four described seasons for South Africa (Southern Hemisphere) and these are summer (December–February), autumn (March–May), winter (June–August) and spring (September–November). During the summer months, temperatures range from 23 °C to 33 °C, with Durban having the highest average temperatures. For both regions January is the hottest month, with June being the coldest month.

Descriptive and inferential statistics were used to evaluate the difference in HbA1c values over the 5-year period for all samples and for patient groups, > 40 years, ≤ 40 years, females and males using IBM^®^ Statistical Package for Social Sciences (SPSS) and Microsoft Excel 365, and a *p*-value < 0.05 was considered statistically significant. Participant characteristics are shown in Appendix 1 together with the group statistics for HbA1c and eAG. Descriptive statistics were performed on the data, and the Kolmogorov–Smirnov test for normality was used for age, eAG and HbA1c. None of the three parameters had normally distributed data (*p* < 0.01), which leads to rejection of the null hypothesis that the data were normally distributed.

The *t*-test for equality of means was used to evaluate the differences in the means between the winter (cold) and summer (warmer) months for HbA1c and eAG.

Table 1 shows the distribution of population and yearly frequencies between the two centres and Table 2 shows the distribution of the population and yearly frequencies between the two centres.

**TABLE 1:** Distribution of population and yearly frequencies between the two centres.

Location	Frequency (*n*)	%	Valid percent age (%)	Reviewed date categorical yearly
NHLS-IALH	62 052	74.1	74.1	-
NHLS-TAD	21 668	25.9	100.0	-
Total	83 720	100.0	-	2016
NHLS-IALH	89 495	78.8	78.8	-
NHLS-TAD	24 107	21.2	100.0	-
Total	113 602	100.0	-	2017
NHLS-IALH	93 430	75.1	75.1	-
NHLS-TAD	31 054	24.9	100.0	-
Total	124 484	100.0	-	2018
NHLS-IALH	94 711	72.5	72.5	-
NHLS-TAD	35 996	27.5	100.0	-
Total	130 707	100.0	-	2019
NHLS-IALH	84 329	66.2	66.2	-
NHLS-TAD	42 987	33.8	100.0	-
Total	127 316	100.0	-	2020

NHLS-IALH, National Health Laboratory Services-Inkosi Albert Luthuli Hospital; NHLS-TAD, National Health Laboratory Services-Tshwane Academic Division.

**TABLE 2:** Distribution of population and yearly frequencies between the two centres.

Group statistics	Cold_warm_monthly_codes	*n*	Mean	s.d.	s.e. Mean
% HbA1c	Colder months	196 233	8.61	2.92	0.0066
	Warmer months	383 596	8.55	2.91	0.0047
EAG	Colder months	196 233	12.75	5.78	0.0131
	Warmer months	383 596	12.64	5.77	0.0093

HbA1c, glycated haemoglobin; EAG, estimated average glucose; s.d., standard deviation; s.e., standard error.

### Ethical considerations

This study was approved by the University of Pretoria Faculty of Health Sciences Research Ethics Committee (Ethics Reference No.: 591/2020) in accordance with the Declaration of Helsinki.

## Results

There was a statistically significant increase (Table 2) in both mean HbA1c and mean eAG in the colder months (May–August) when compared to the warmer months (November–February) over the 5-year period (*p* < 0.01). There was also a significant difference in the eAG in the same 5-year period for warmer months and colder months (*p* < 0.01).

**FIGURE 1:**
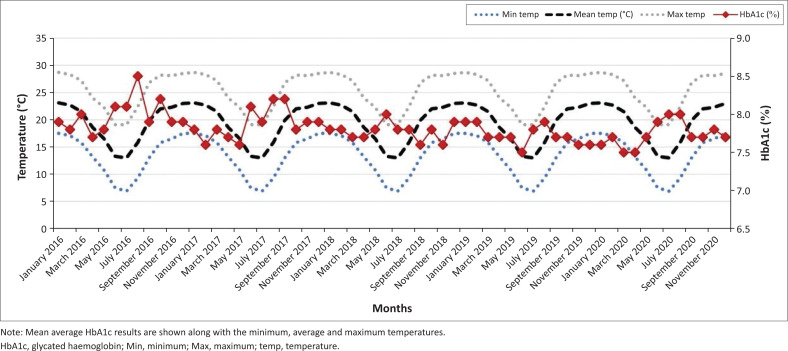
Seasonal variation in mean glycated haemoglobin results over a 5-year period (Gauteng province).

**FIGURE 2:**
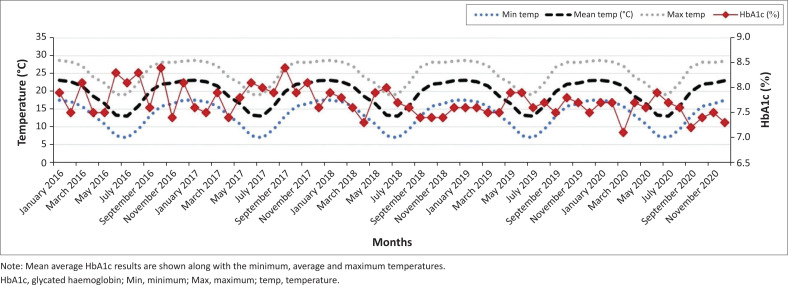
Seasonal variation in mean glycated haemoglobin results over a 5-year period (KwaZulu-Natal province).

**FIGURE 3:**
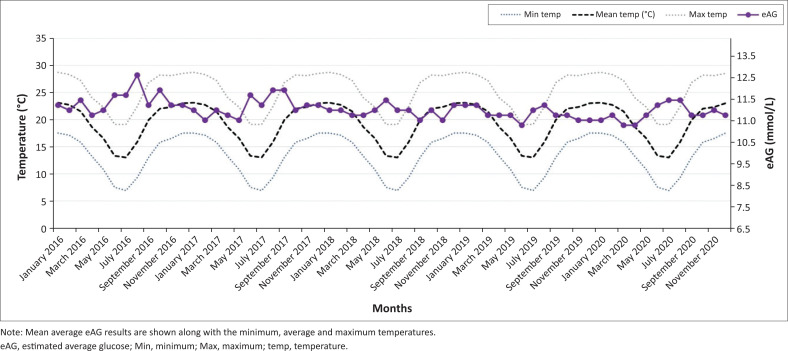
Seasonal variation in mean estimated average glucose results over a 5-year period (Gauteng province).

**FIGURE 4:**
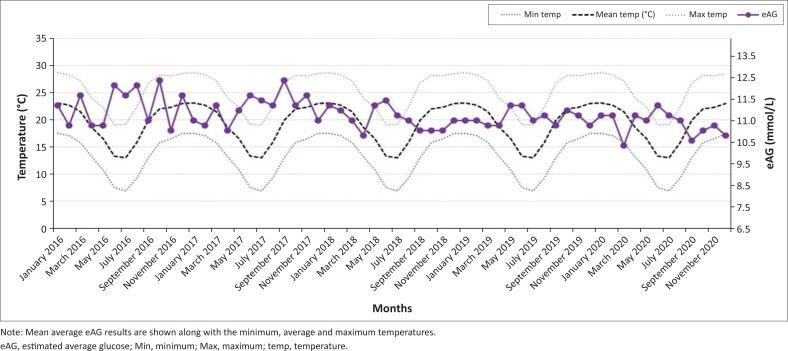
Seasonal variation in mean estimated average glucose results over a 5-year period (KwaZulu-Natal province).

A good correlation (*R*-squared value of 0.958) was observed between the mean eAG and mean HbA1c, which further affirmed the statistically significant difference in the summer and winter months for both parameters. Figure 1 shows the mean glycated haemoglobin (HbA1c) for each month of the year in the Gauteng province (January 2016 – December 2020; *p* < 0.01). There was a significant increase in mean HbA1c in the winter months (May, June, July) when compared to the summer months (December, January, February) in the 5-year period. There was no significant difference in HbA1c between the summer months of consecutive years (e.g., November 2016 and November 2017) or the winter months of consecutive years (e.g., June 2019 vs. June 2020). Figure 2 shows mean glycated haemoglobin (HbA1c) for each month of the year in the KwaZulu-Natal province (January 2016 – December 2020; *p* < 0.01). A similar but less pronounced trend is observed in the warmer province with peak HbA1c in the colder months. This is noticeable when comparing January 2019 and July 2020 (*p* < 0.01). There was no significant difference in HbA1c between summer months of consecutive years as observed in the Gauteng province. Figure 3 and Figure 4 demonstrate the eAG for each month of the year for Pretoria and Durban, respectively (January 2016 – December 2020; *p* < 0.01), along with mean temperatures. The distribution of the Cohen’s d statistic was also calculated to evaluate the effect size of the hypothesis that the HbA1c and eAG means were significantly different between colder months and warmer months. A value greater than 0.8 (unitless) indicates that the means are significantly different. The value obtained for all the data was 2.9174, exceeding the threshold of 0.8 for a good effect size. When the mean HbA1c values were further divided for age groups and gender, there was still a statistically significant difference between the means of the winter and summer means (*p* < 0.01) with a Cohen’s d effect size > 0.8.

## Discussion

The Direct Control and Complications Trial (DCCT) using HPLC demonstrated a direct relationship between HbA1c and the development of complications.^15^ The success of this study led to the need to align HbA1c measurements to patient outcomes. The NGSP was formed to standardise HbA1c methods such that results from different laboratories would be comparable to those of the DCCT.^15^

The IFCC-WG approved HPLC and electrospray ionisation mass spectrometry as the reference methods for the quantitation of HbA1c in human blood.^1,16,17^ This working group recommended that all laboratories should only use NGSP-certified assays and use both NGSP and DCCT units when reporting results. Capillary electrophoresis and HPLC with ultraviolet (UV) detection are also used and yield comparable results.^15,17^

Today HbA1c testing is used internationally to diagnose and monitor patients with diabetes mellitus. It is recommended that a 6-month testing frequency be considered for patients meeting treatment goals and shorter intervals in those for whom glycaemic control is not yet achieved.^18^ In time-series analyses of data when evaluating seasonality of an analyte, it is important to know its clinical utility, characteristics and the adequacy of its analytical methods by reporting its performance statistically.^13,19^

Studies done in the northern hemisphere demonstrated seasonal variations of HbA1c, showing values to be higher during cooler seasons (autumn and winter) than during warmer months (spring and summer).^2,6,7^ HbA1c represents glycaemic control for the past 3 months. Higher values indicate poor glycaemic control for that period and may miss patients who have good control of their plasma glucose at the exact time of testing.

‘Big data’ analysis has been used in several cross-sectional studies to reveal seasonal variation in multiple other analytes such as potassium, cholesterol and vitamin D.^19,20^ Patients may be diagnosed as diabetic when in fact they may be pre-diabetic based on when they are tested.^7^ Some pre-diabetic patients may also be missed when tested in summer months as opposed to when tested in winter months when higher values of HbA1c are seen. This variation may also influence dosing patterns during an individual’s treatment monitoring.

The population in this study has unique qualities in terms of geographical location, mean temperatures, socioeconomic status and dietary differences. This could lead to adverse effects such as hypoglycaemia when patients exhibit better control in summer months.^21^

This study demonstrated seasonal variation in both HbA1c and eAG for both provincial regions, the combined data (Durban and Pretoria) and for the different categories of patients based on gender and age.

## Conclusion

This study provides useful information regarding seasonal variation of HbA1c and eAG in an African population. Being aware of this seasonality may assist clinicians in treatment planning, adjustment of testing frequencies and establishing specific HbA1c testing time points to avoid over- or underdosing of medication. Further local studies of this nature will provide an opportunity for revision of local guidelines with respect to testing frequencies, time points in a year when patients should be tested and the adjustment of treatment targets and diagnostic cutoffs to prevent adverse effects.

## Appendix 1

**TABLE 1-A1:** Participant characteristics and yearly frequencies are depicted for age with group statistics for each age group.

Age categorical (years)	Frequency	%	Valid percent (%)	Cumulative percent
**2016**				
0–40	11 274	13.5	13.5	13.5
> 40	72 446	86.5	86.5	100.0
Total	83 720	100.0	100.0	-
**2017**				
0–40	13 964	12.3	12.3	12.3
> 40	99 638	87.7	87.7	100.0
Total	113 602	100.0	100.0	-
**2018**				
0–40	16 244	13.0	13.0	13.0
> 40	108 240	87.0	87.0	100.0
Total	124 484	100.0	100.0	-
**2019**				
0–40	17 184	13.1	13.1	13.1
> 40	113 523	86.9	86.9	100.0
Total	130 707	100.0	100.0	-
**2020**				
0–40	18 039	14.2	14.2	14.2
> 40	109 277	85.8	85.8	100.0
Total	127 316	100.0	100.0	-

**TABLE 1-A2:** Participant characteristics and yearly frequencies are depicted for age with group statistics for each age group.

Group statistics	Age categorical (years)	*n*	Mean	s.d.	s.e.
% HbA1c	0–40	76 705	8.45	3.52	0.01
	> 40	503 124	8.59	2.81	0.00
eAG	0–40	76 705	12.44	6.96	0.02
	> 40	503 124	12.71	5.57	0.01

HbA1c, glycated haemoglobin; EAG, estimated average glucose; s.d., standard deviation; s.e., standard error.

**TABLE 1-A3:** Participant characteristics and yearly frequencies are depicted for gender with each gender’s group statistics.

Gender	Frequency	%	Valid percent (%)	Cumulative percent
**2016**				
Female	56 506	67.5	67.5	67.5
Male	27 214	32.5	32.5	100.0
Total	83 720	100.0	100.0	-
**2017**				
Female	77 184	67.9	67.9	67.9
Male	36 418	32.1	32.1	100.0
Total	113 602	100.0	100.0	-
**2018**				
Female	83 736	67.3	67.3	67.3
Male	40 748	32.7	32.7	100.0
Total	124 484	100.0	100.0	-
**2019**				
Female	87 676	67.1	67.1	67.1
Male	43 031	32.9	32.9	100.0
Total	130 707	100.0	100.0	-
**2020**				
Female	84 190	66.1	66.1	66.1
Male	43 126	33.9	33.9	100.0
Total	127 316	100.0	100.0	-

**TABLE 1-A4:** Participant characteristics and yearly frequencies are depicted for gender with each gender’s group statistics.

Group statistics	Gender	*n*	Mean	s.d.	s.e.
% HbA1c	Female	389 292	8.66	2.904	0.00
	Male	190 537	8.38	2.93	0.01
EAG	Female	389 292	12.86	5.75	0.01
	Male	190 537	12.31	5.81	0.01

HbA1c, glycated haemoglobin; EAG, estimated average glucose; s.d., standard deviation; s.e., standard error.
